# Extending geriatric care beyond traditional settings: lessons from a mobile assessment program

**DOI:** 10.1186/s13584-026-00775-y

**Published:** 2026-08-10

**Authors:** Zorian Radomyslsky, Dor Atias, Bar Cohen, Ali Abu-Raya, Sara Kivity, Reuma Kurz, Eduardo Schejter, Jacob Segal, Ilan Yehoshua, Ori Liran, Miri Mizrahi Reuveni, Galit Kaufman, Daniela Rahamim-Cohen, Limor Adler

**Affiliations:** 1 Maccabi Healthcare Services, Tel Aviv, Israel; 2 School of Health Sciences, Ariel University, Ariel, Israel; 3 Department of Epidemiology and Preventive Medicine, School of Public Health, Gray Faculty of Medical & Health Sciences, Tel Aviv University, Tel Aviv, Israel; 4 Gray’s Faculty of Medical & Health Sciences, Tel Aviv University, Tel Aviv, Israel; 5 Department of Family Medicine, Gray’s Faculty of Medical & Health Sciences, Tel Aviv University, Tel Aviv, Israel; 6 Dina Recanati School of Medicine, Reichman University, Herzliya, Israel; 7 Bait Balev, Tel Aviv, Israel

**Keywords:** Geriatric Assessment, Accessibility, Mobile Geriatric Team, Elderly Care

## Abstract

**Background:**

As populations age, changing living conditions and declining self-advocacy often create barriers to essential care. Mobile Geriatric Teams (MGTs) offer a potential solution by providing assessments within a patient’s regular environment, potentially reaching underdiagnosed and undertreated individuals who struggle to access routine healthcare. The aim of this study was to evaluate the clinical impact and reach of MGTs in the Northern District of Israel. Specifically, we aimed to assess the extent of MGT-initiated Comprehensive Geriatric Assessments and compare therapeutic and diagnostic outcomes between patients seen by MGTs and those receiving standard community-based geriatric consultations.

**Methods:**

This is a retrospective, data-based study serving as a proof-of-concept evaluation for MGT implementation in Israel. The intervention was implemented in MHS between January 1, 2020, and December 31, 2023, and data were collected 3 and 6 months after the MGT appointment. Group comparisons were conducted using chi-square tests, Fisher’s exact tests, and Wilcoxon rank-sum tests. In addition, we performed matched logistic regression models.

**Results:**

A total of 8,152 individuals were included in the study; 4,348 were assessed by MGTs. The MGT group participants were slightly older and had a higher proportion of males and a lower SES category than the comparison group. The MGTs group had fewer diagnoses of mild cognitive impairment, dementia, and depression, compared to the comparison group. They also had more diagnoses of orthostatic hypotension, and they were more likely to be assessed for fall risk. The MGT group was also prescribed fewer medications in the months following the assessment. After the MGT intervention, we report an increase in primary care visits in the relevant population in the district where it was implemented, while the comparison group did not have a corresponding trend.

**Conclusions:**

In this study, we demonstrated that MGTs can reach a specific subpopulation of the elderly who might not otherwise undergo a geriatric assessment, primarily males, those of low SES, and possibly those with less urgent health concerns. These findings highlight the complementary role of MGTs in increasing accessibility, promoting equity, and addressing unmet needs in populations less likely to seek care on their own.

## Background

Rapid population aging presents a significant challenge to global healthcare systems. While Israel’s population is currently younger than the OECD average (12.4% aged 65+), it is facing a rapid demographic shift that demands a proactive reallocation of healthcare resources [1]. Aging is associated with an increased prevalence of chronic morbidity and a decline in functional independence (ADL/IADL), complicating care delivery [2]. In response, a 2021 Israeli government mandate prioritized early risk detection and the prevention of chronic morbidity, with a specific focus on sociodemographic factors to improve quality of life [3].

Comprehensive geriatric assessment is a multidisciplinary diagnostic process used to evaluate the medical, psychological, and functional status of elderly individuals [4, 5]. Emerging evidence suggests that community-based comprehensive geriatric assessments can improve functional status and quality of life, while also reducing mortality and hospitalization [6, 7]. One of the primary challenges in exploring the potential benefits of such an assessment in the community is the elderly population’s general barriers to healthcare services. While the elderly in general use more healthcare services and have greater health-related needs, many encounter obstacles, including limited mobility, financial constraints, difficulty navigating administrative processes, and restricted access to transportation and appropriate care providers [8, 9]. These challenges are compounded by systemic barriers such as limited access to relevant professionals, long waiting times, short appointments, and environments that are not suited to their limitations [10].

**Fig. 1:**
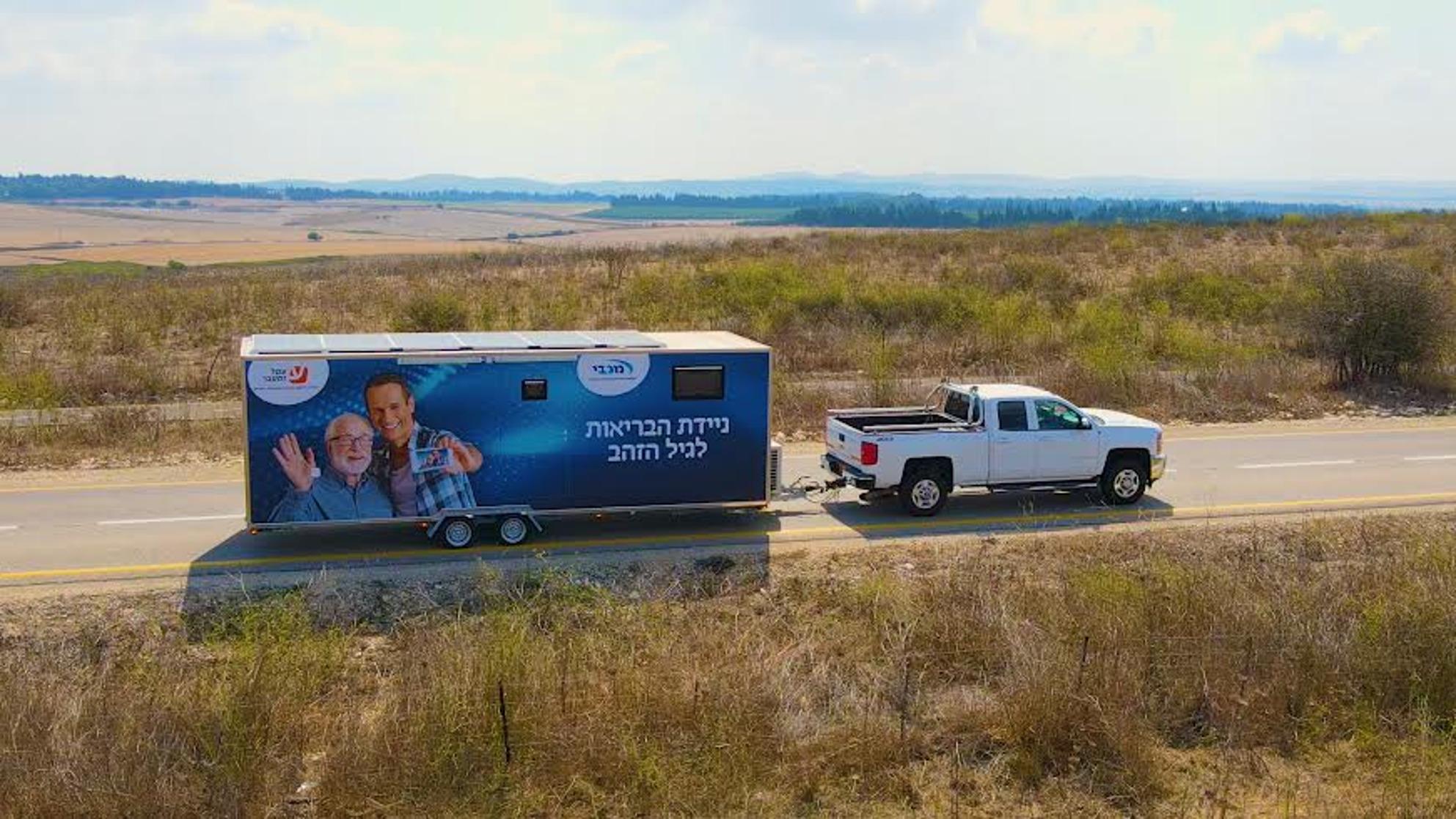
A photograph of Maccabi Healthcare Services’ Mobile Geriatric Team on the roads of the North district of Israel

**Fig. 2:**
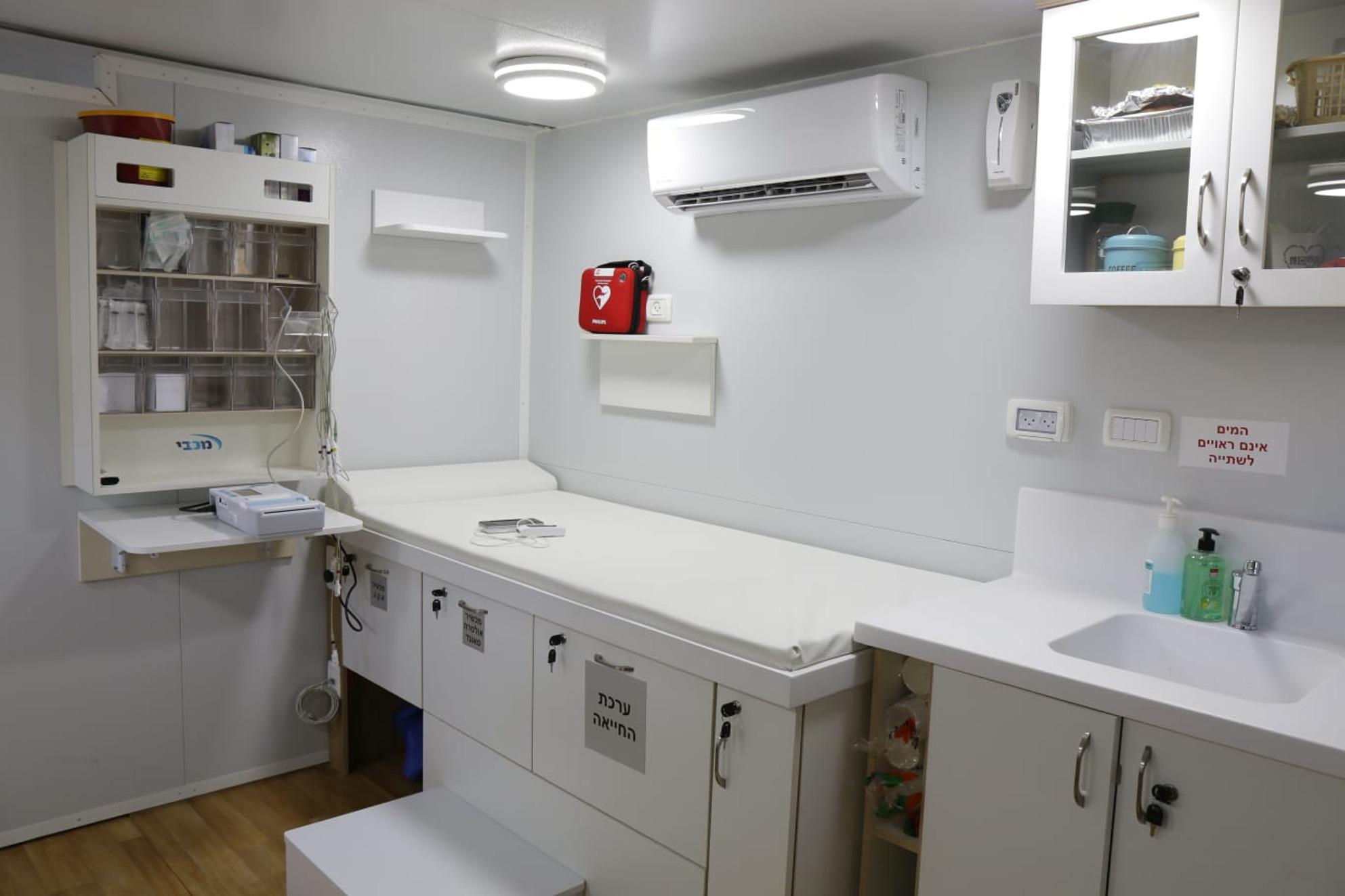
A photograph of the Interior view of the Maccabi Healthcare Services Mobile Geriatric Team Clinic

13% of Maccabi Healthcare Services (MHS) members are over 65, with this proportion rising to 18% in the Northern district of Israel. This region not only has a larger elderly population but is also characterized by low population density, with many residents living in small, remote locations. Consequently, access to specialized healthcare services often requires substantial travel. To address these unique regional needs, the Geriatric Department at MHS launched a mobile geriatric team (MGT) to reach older adults who struggle to access routine healthcare (see Figs. 1 and 2) [11]. Patients were proactively identified and invited to be evaluated at the MGT based on MHS administrative data; those with borderline eligibility or low levels of entitlement under the National Long-Term Care Insurance Law, and those living with chronic conditions.

The outreach process was carried out in collaboration with local municipalities to ensure comprehensive community engagement. Outreach was conducted after all primary care physicians were briefed on the program’s objectives; patients were excluded only if their physician actively opted out of the initiative. Eligible individuals were then contacted directly by phone by a dedicated administrative coordinator. Regional visibility was further supported by advertisements on the MHS website and within local municipal frameworks.

The mobile clinic team consisted of a geriatrician, a nurse, and an occupational therapist, with additional support from a social worker at the local branch when needed. To maximize accessibility, the MGT was stationed at central locations within the municipalities, such as senior day centers or commercial hubs. Each municipality committed to providing a site with essential utility hookups (water and electricity) and level ground (zero-grade) for safe operation. While MHS requested the presence of a municipal representative to facilitate continuity of care within the community, this role was not consistently filled due to limited responsiveness from local authorities.

In this study, we aimed to assess the reach and extent of an MGT-initiated comprehensive geriatric assessment for elderly patients living in the Northern district of Israel and to compare the therapeutic and diagnostic outcomes of this population with those of patients who attended regular geriatric consultations in the community.

## Methods

### Study design and setting

This retrospective, data-based study serves as a proof-of-concept evaluation for the MGT intervention implemented in MHS between January 1, 2020, and December 31, 2023. We compared patients assessed by the MGT with counterparts assessed by geriatricians in regular clinics (the standard care). Notably, given that both groups are in the same district, the resources and structure of healthcare services are presumably similar. So, in essence, the professionals involved were similar in terms of their characteristics. The study was approved by the MHS ethical committee (#0129-23-MHS).

## Participants

The intervention (case) group included MHS members aged 65 and older who underwent a comprehensive geriatric assessment by the MHS MGT during the study period in the northern district. The comparison group consisted of similarly aged MHS members who underwent a comprehensive geriatric assessment in a traditional setting from the northern district of Israel (i.e., those who independently initiated a visit to a geriatric specialist). These criteria were chosen to determine whether the characteristics of those using the MGT service differed at baseline.

## Variables

We extracted sociodemographic variables, including gender, socioeconomic status (SES), and age. The SES category for each individual was documented in the MHS database, originally obtained from the Central Bureau of Statistics based on the individual’s exact address. It was then categorized, for statistical purposes, into three groups: low [1–4], middle [3–7], and high [8–10].

The outcome diagnoses were the number of primary care visits, visits to other healthcare providers, level of home assistance required (according to the Israeli Nursing Law, instituting specific levels of disability entitling the patients to assistance), new mild cognitive impairment or dementia diagnosis (based on recorded diagnoses in the electronic medical record), fall assessments (based on standardized MHS protocol), memory loss medication prescribed and purchased (prescription fulfilled), newly submitted depression diagnosis, anti-depressants prescribed and purchased (prescription fulfilled), and orthostatic blood pressure diagnosis (recorded in electronic medical recorded, and based on accepted orthostatic hypotension measurements). For medications, we examined prescribing and purchasing as independent metrics, regardless of diagnosis, to assess their implications for adherence and subjective need. We re-examined and evaluated trends three and six months after the intervention, coinciding with quarterly intervals during which care and services are usually examined and discussed at MHS and in all other local HMOs. Our data is based on metrics documented in the electronic health record and does not include the free-text segment of the letter and appointment summary. Naturally, not all physicians chart similarly; however, these metrics are needed for prescribing and other actions and are therefore likely to be more accurate than free text.

### Statistical analysis

Categorical variables are reported as counts and percentages, and continuous variables are reported with means and standard deviations. Group comparisons were conducted using chi-square tests, Fisher’s exact tests, and Wilcoxon rank-sum tests, as appropriate. Additionally, we compared the total number of primary care or specialized geriatric visits among MHS members aged 65 + across the Northern and Southern districts to provide a fuller view of the intervention’s overall effect and to establish that the intervention did not hinder and fragment community care.

To account for baseline differences between groups, we performed a propensity score–matched analysis. The propensity score for receiving MGT assessment was estimated using age, sex, and SES among participants from the Northern district. Participants were matched 1:1 using nearest-neighbor matching with a caliper of 1.5. Covariate balance before and after matching was evaluated using standardized mean differences.

We then examined four binary outcomes: Dementia/mild cognitive impairment diagnosis at visit, depression diagnosis at visit, fall assessment performance in three months after the visit, and Elevation in nursing care level, 6 months after the visit. For each outcome, we fitted a conditional logistic regression model stratified by matched pair (subclass), with additional adjustment for age, sex, and SES to account for any residual imbalance. Results are reported as odds ratios (ORs) with 95% confidence intervals (CIs). All tests were two-sided, and *p* < 0.05 was considered statistically significant.

## Results

### Participants

A total of 8,152 individuals were included in the study, 4,348 in the case group (i.e., assessed by the MGT) and 3,804 in the comparison group (i.e., assessed by a geriatric specialist in a traditional setting).

## Descriptive data

On average, the case group was slightly older than the comparison group, with a mean age of 76.4 years compared to 76.1 years (p-value = 0.001). The case group had a lower proportion of females than the comparison group (53% vs. 58%; p-value < 0.001). The two groups varied greatly in SES characteristics (44% vs. 28% in the low-SES group and 8.7% vs. 27% in the high-SES group in the case and comparison groups, respectively; p-value < 0.001) (Table 1).

**Table 1:** Baseline Characteristics and Follow-Up Outcomes of Patients Receiving Mobile Geriatric Team (MGT) Services Compared to Standard Geriatric Assessment

Characteristic	Overall *N* = 8,152^1^	MGT group *N* = 4,348^1^	Comparison Group *N* = 3,804^1^	*p*-value^3^
Age at visit	76.3 (5.7)	76.4 (4.7)	76.1 (6.6)	0.001
Sex (Female)	4,517 (55%)	2,323 (53%)	2,194 (58%)	< 0.001
Socioeconomic status				
Low	2,985 (37%)	1,934 (44%)	1,051 (28%)	< 0.001
Medium	3,742 (46%)	2,035 (47%)	1,707 (45%)	
High	1,425 (17%)	379 (8.7%)	1,046 (27%)	
Performance of the Orthostatic blood pressure test during the visit	162 (2.0%)	115 (2.6%)	47 (1.2%)	< 0.001
Receiving a diagnosis of Dementia or mild cognitive impairment during the visit	822 (10%)	389 (8.9%)	433 (11%)	< 0.001
Depression after the visit	302 (3.7%)	134 (3.1%)	168 (4.4%)	0.001
Fall assessment in 3 months after visit	586 (7.2%)	426 (9.8%)	160 (4.2%)	< 0.001
Physiotherapy visits (n)	2.6 (6.1)	2.3 (5.4)	2.8 (6.8)	> 0.9
Prescribed memory-loss medications at the visit	381 (4.7%)	68 (1.6%)	313 (8.2%)	< 0.001
Purchased memory-loss medications at the visit	289 (3.5%)	51 (1.2%)	238 (6.3%)	< 0.001
Purchased depression medications 3 months after the visit	187 (2.3%)	39 (0.9%)	148 (3.9%)	< 0.001
Purchased any medications in the 3 months after the visit	6,611 (81%)	3,356 (77%)	3,255 (86%)	< 0.001
Nursing care level at visit				
None	6,484 (80%)	3,385 (78%)	3,099 (81%)	< 0.001
1–2	1,189 (15%)	683 (16%)	506 (13%)	
3	479 (5.9%)	280 (6.4%)	199 (5.2%)	
4–6	0 (0%)	0 (0%)	0 (0%)	
Nursing care level, 3 months after the visit				
0	5,995 (74%)	3,242 (75%)	2,753 (72%)	0.028
1–2	1,378 (17%)	723 (17%)	655 (17%)	
3	779 (9.6%)	383 (8.8%)	396 (10%)	
4–6	0 (0%)	0 (0%)	0 (0%)	
Elevation in nursing care level, 3 months after visit	749 (9.2%)	257 (5.9%)	492 (13%)	< 0.001
Nursing care level, 6 months after the visit				
0	5,853 (72%)	3,158 (73%)	2,695 (71%)	0.065
1–2	1,454 (18%)	770 (18%)	684 (18%)	
3	845 (10%)	420 (9.7%)	425 (11%)	
4–6	0 (0%)	0 (0%)	0 (0%)	
Elevation in nursing care level, 6 months after visit	987 (12%)	382 (8.8%)	605 (16%)	< 0.001

## Main results

Significant differences were observed in clinical outcomes and health services utilization between the groups. For convenience, we report percentages to better convey our findings; however, all absolute numbers are available in the Tables. The case group had more diagnoses of orthostatic hypotension at the visit than the comparison group (2.6% vs. 1.2%, p-value < 0.001). Additionally, the case group had a higher proportion of fall assessments in the three months after the visit than the comparison group (9.8% vs. 4.2%, p-value < 0.001) (See Table 1).

The case group had fewer diagnoses of dementia or mild cognitive impairment at the visit than the comparison group (8.9% vs. 11%, p-value < 0.001). Correspondingly, fewer patients in the case group were prescribed memory loss medications during the visit or purchased them after the visit than in the comparison group (1.6% vs. 8.2% and 1.2% vs. 6.3%, respectively; p-value < 0.001 for both).

Fewer patients in the case group were diagnosed with depression during the visit compared with the comparison group (3.1% vs. 4.4%, p-value = 0.001), and purchased anti-depressants in the three months following the visit compared with the comparison group (0.9% vs. 3.9%, p-value < 0.001). Overall, a smaller proportion of case group patients purchased any prescription medication in the three months following the visit compared with the comparison (77% vs. 86%, p-value < 0.001).

**Fig. 3:**
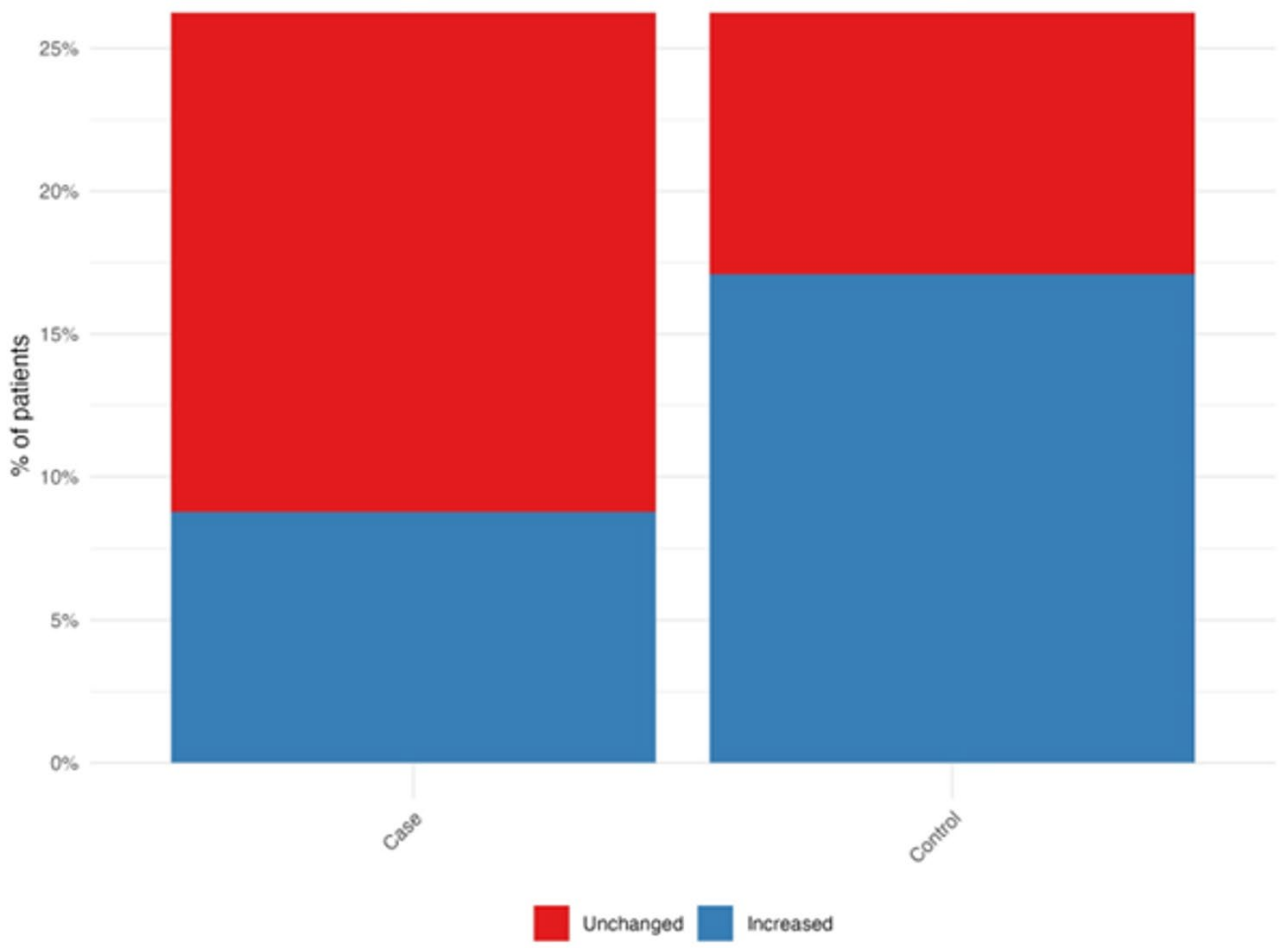
Change in assistance eligibility level within six months post-assessment. Stacked bar chart showing the proportion of patients whose level of assistance eligibility increased (blue) or remained unchanged (red) in the case (MGT) and comparison groups

Three months after the visit, proportions of patients not entitled to home assistance was statistically significantly different between groups (75% in the case group vs. 72% in the comparison group, p-value = 0.028), but the absolute difference was small and may not be clinically meaningful. This pattern persisted for 6 months after the original visit (73% in the case group and 71% in the comparison group; p-value = 0.065). However, within three months after the visit, a greater proportion of patients in the comparison group were assigned a higher level of assistance eligibility (13% vs. 5.9%, p-value < 0.001). Overall, the comparison group exhibited a higher rate of change in the recorded level of assistance eligibility during the follow-up period (See Fig. 3; Table 1).

After matching, 3,232 matched pairs were included in the analysis, with all absolute standardized mean differences below 0.10 (Table 2). Participants assessed by the MGT had lower odds of being diagnosed with dementia/ mild cognitive impairment than comparison participants (OR 0.64, 95% CI 0.53–0.76; *p* < 0.001). They also had lower odds of depression, although the association was borderline and did not meet statistical significance (OR 0.77, 95% CI 0.58–1.02; *p* = 0.072). By contrast, MGT participants had higher odds of undergoing a fall assessment (OR 2.40, 95% CI 1.86–3.10; *p* < 0.001). They also had lower odds of improvement in nursing care level at 6 months (OR 0.38, 95% CI 0.32–0.45; *p* < 0.001) (Table 3).

**Table 2:** Covariate balance before and after matching

Covariate	All data GMT cohort	All data Control cohort	All data SMD	Matched GMT cohort	Matched Control cohort	Matched SMD
Age, years	76.13	76.41	-0.043	75.94	76.48	-0.083
Female sex, %	57.7	53.4	0.086	62.6	58.0	0.092
Low SES, %	27.6	44.5	-0.377	32.5	32.6	-0.003
Medium SES, %	44.9	46.8	-0.039	52.8	55.6	-0.057
High SES, %	27.5	8.7	0.421	14.7	11.7	0.066

**Table 3:** Matched conditional logistic regression analysis. Odds ratios were derived from conditional logistic regression models stratified by matched subclass and additionally adjusted for age, sex, and SES

Outcome	Odds ratio	95% CI	*P* value
Dementia or mild cognitive impairment	0.64	0.53–0.76	< 0.001
Depression	0.77	0.58–1.02	0.072
Fall assessment	2.40	1.86–3.10	< 0.001
Elevation in nursing care level	0.38	0.32–0.45	< 0.001

**Fig. 4:**
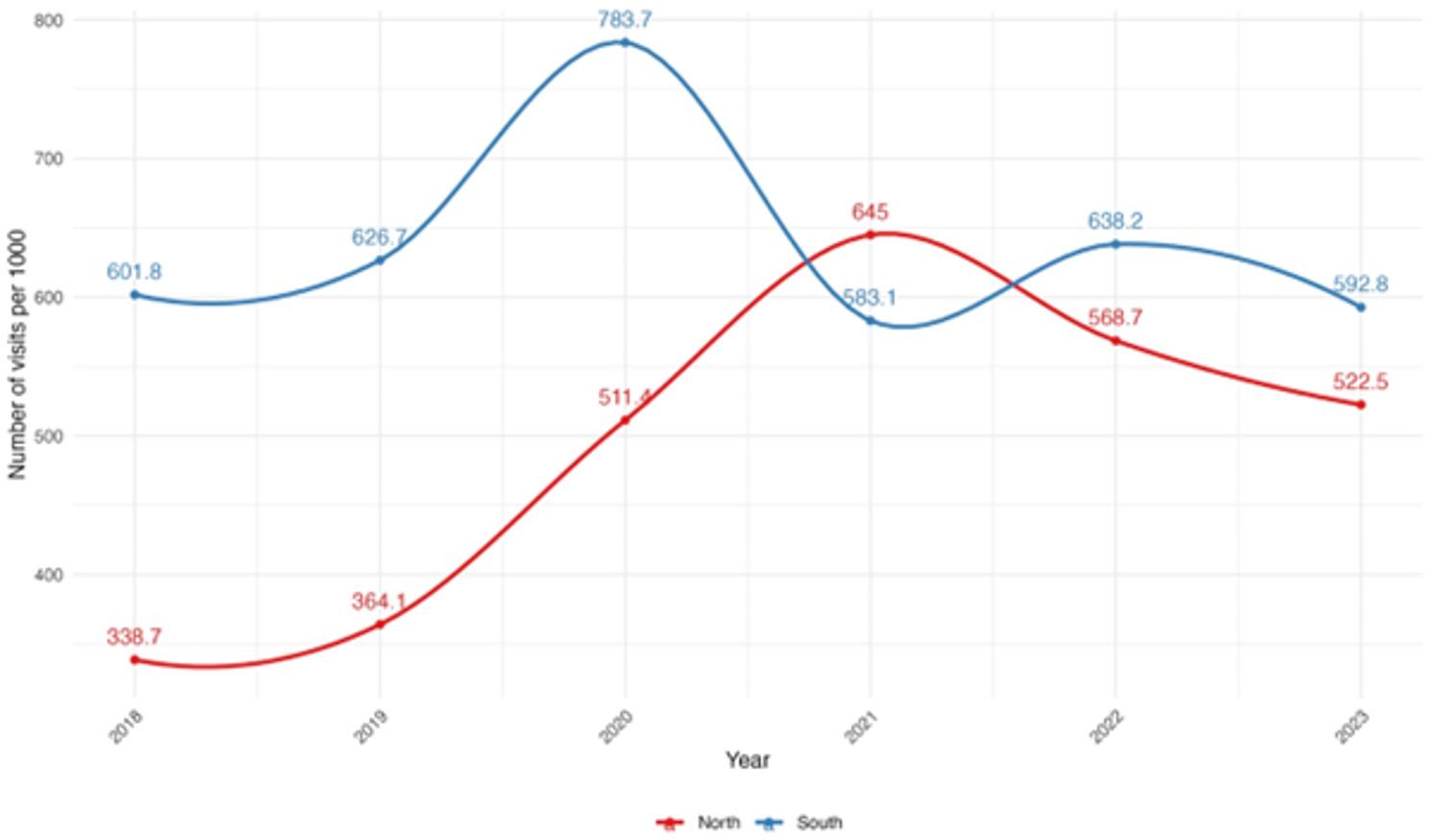
Trends in medical visits per 1,000 individuals aged 65 and older, by district. Line graph comparing the number of annual visits to primary and specialized care per 1,000 elderly individuals in the Northern (red) and Southern (blue) districts

Before the intervention, the Northern district, despite having a higher proportion of elderly residents, had substantially fewer visits to primary care and specialized medical services than the Southern district. Around 2021, however, this trend changed, with a significant increase in visits in the Northern district, coinciding with the peak implementation of the MGT intervention. These visits include both MGT visits and follow-up visits to specialists and primary care physicians after the initial MGT visit (See Fig. 4). Moreover, this change happened during the COVID-19 pandemic, which disrupted the healthcare services provided in community settings, especially for the elderly, who were perceived as especially vulnerable.

## Discussion

### Main results

This retrospective, data-based study evaluated the implementation of the MGT project in Northern Israel (2020–2023). Our analysis reveals two primary outcomes significant to the Israeli healthcare landscape. First, the MGT reached a distinct demographic compared to traditional clinic-based services: participants were significantly more likely to belong to a low-SES group and included a higher proportion of males. Second, while the MGT group had fewer pre-existing chronic diagnoses and conditions at presentation (such as dementia, depression, and complex polypharmacy), we report a marked increase in proactive screening outcomes in this group, specifically regarding orthostatic hypotension detection and subsequent fall risk assessments.

### Interpretation

Our findings suggest that the MGT model serves as a proactive outreach mechanism that successfully addresses the shortcomings or discrepancies of more traditional settings. In the context of Israel’s universal healthcare system, where clinical appointments are freely offered and covered, our data identifies that financial constraints remain a formidable barrier [12]. The over-representation of low-SES individuals in the MGT cohort suggests that “indirect costs” may affect the consumption of healthcare services, especially those not acutely needed. Such indirect costs include the need for a caregiver escort, transportation logistics in the periphery, or the inability to miss work [8, 9]. Notably, we did not measure these costs, and our findings may be attributed to service availability, accessibility, or quality; however, we believe indirect costs should also be taken seriously as potential barriers.

Furthermore, the clinical disparity between the two groups reflects a difference in health-seeking behaviors. While clinic-based patients are often driven by an existing symptomatic burden (e.g., memory loss or mood changes), the MGT successfully engages a “silent” population, who are ideal for screening efforts in addition to treatment efforts. These are individuals who may not perceive their symptoms as significant enough to warrant a clinic visit [13] or those who lack the social support to initiate one. The higher detection rate of orthostatic hypotension and the surge in fall assessments in the MGT group underscore the project’s efficacy in shifting from reactive treatment to the detection of silent risk factors for morbidity.

### Implications

The results of this proof-of-concept study have direct implications for geriatric service delivery in Israel:

#### Promoting Health Equity

The MGT model can serve as a potential tool to reduce health disparities. By moving the point of care to the home, the healthcare system can perhaps bypass some socioeconomic barriers that currently limit the reach of the National Health Insurance Law for marginalized populations. This notion is supported by the differences found between the populations, notably that the MGT served more low-SES males.

#### Integrating Specialist and Primary Care

The observed increase in primary care engagement following MGT intervention suggests that mobile teams do not disrupt or fragment care. We can hypothesize that they act as a “gateway” that reintegrates isolated elderly patients into the longitudinal care of their family physicians.

#### National Scalability

As the Israeli population ages, the MGT model can provide a scalable framework for preventive geriatrics. Policy can focus on regulating such teams within HMOs to ensure that geriatric expertise is distributed based on community risk and need and to reach populations underrepresented in other community healthcare settings.

### Strengths and limitations

This study has several notable strengths. First, it includes a comparison between two relevant populations, highlighting the potential use of MGTs to reach underdiagnosed and undertreated populations. Second, we were able to follow up and see the real-world consequences of the intervention in multiple aspects of health and life. Third, we were able to characterize the needs of the populations and prove the usability and sustainability of the MGTs model.

However, this study also has several limitations. First, while we were able to follow up over time, the follow-up was limited to 6 months. It is possible that other advantages or disadvantages of the method only become apparent over longer periods. Second, we did not examine the interactions between said intervention and hospital-based services, such as hospitalizations and ER visits. These services are particularly important for this age group. Additionally, we did not explore the specific reasons for the differences in the characteristics of the case and comparison populations. Specifically, the case group was drawn from the Northern district via proactive outreach by the MGT, whereas the comparison group comprised Northern district patients who self-initiated geriatric clinic visits. These differences may influence characteristics and outcomes independent of the MGT. Additionally, we did not measure hospitalizations, actual falls, mortality, morbidity, and quality-of-life measures in the aftermath of the assessment. These are important geriatric outcomes that could have enhanced this study. In the future, we suggest a long-term combined follow-up, including data from hospitals and community healthcare services. Finally, so far, the program has been conducted only in a specific time and place as a proof-of-concept effort. These limitations should be addressed by research endeavors in general and potentially by a more extensive implementation of the discussed program.

## Conclusions

In this study, we demonstrated that implementing an MGT can reach a specific subpopulation of the elderly who might otherwise remain underserved by traditional clinic-based geriatric assessment: primarily males, those of low SES, and those in the periphery. This population represents a critical target group for preventive care and early screening interventions that align with national health equity goals. In contrast, individuals who independently sought geriatric assessment were more likely to be diagnosed with cognitive impairment and depression and were more frequently prescribed medications. This suggests that while specific clinical concerns or perceived health needs often drive self-initiated engagement, the MGT model successfully identifies “silent” risk factors, such as fall risk, before they escalate. Taken together, these findings highlight the complementary role of MGTs in increasing accessibility, bypassing logistical barriers to the National Health Insurance Law, and addressing unmet needs in populations less likely to seek care on their own. This proactive approach offers a scalable framework for integrating community-based specialized care into the Israeli primary care ecosystem.

## Data Availability

The datasets used and/or analyzed during the current study are not available due to ethical restrictions.

## Abbreviations

MHS: Maccabi Health Services
MGT: Mobile Geriatric Team
SES: Socioeconomic Status
